# Mechanisms restricting hepatitis B virus cross-species transmission

**DOI:** 10.1128/mbio.03763-25

**Published:** 2026-06-18

**Authors:** Jenny N. Ijoma, Andoni Gomez-Moreno, Samer Ayoub, Alexander Ploss

**Affiliations:** 1 Department of Molecular Biology, Princeton University 6740, Princeton, New Jersey, USA; Vallabhbhai Patel Chest Institute, Delhi, India

**Keywords:** hepatitis B, hepatitis B virus, HBV, species tropism, animal model

## Abstract

Chronic hepatitis B virus (HBV) infection affects more than 250 million people worldwide and markedly increases the risk of terminal liver diseases, including liver cirrhosis and hepatocellular carcinoma. Despite its global impact, HBV exhibits a remarkably narrow host range, with natural infection largely restricted to humans and chimpanzees. This stringent tropism has posed a major barrier to the development of experimentally tractable *in vivo* models and has limited our ability to study viral persistence, pathogenesis, and immune control. Over the past decades, substantial progress has been made in identifying the host and viral factors that govern HBV species specificity. Efforts to overcome these constraints have led to the development of genetically engineered systems, the use of surrogate hepadnaviruses that naturally infect other species, and the establishment of humanized liver models that enable infection *in vivo* within a human hepatocyte context. While these approaches have provided important insights, no currently available model fully recapitulates chronic HBV infection in an immunocompetent host. Defining and overcoming the barriers that underlie HBV’s restricted host range remains a central challenge in the field. Addressing this limitation will be essential for establishing physiologically relevant model systems and, ultimately, for enabling the development of curative strategies for chronic hepatitis B.

## INTRODUCTION

## HEPATITIS B VIRUS

Hepatitis B virus (HBV) is a hepatotropic, blood-borne pathogen belonging to the *Hepadnaviridae* family. It remains a major global health burden: more than one-third of the world’s population has been exposed to HBV, with an estimated 257 million individuals living with chronic infection and nearly 887,000 deaths annually attributable to cirrhosis and hepatocellular carcinoma (1–4). Despite the availability of an effective prophylactic vaccine, current antiviral therapies rarely achieve functional cure, necessitating long-term, often lifelong treatment for most chronically infected individuals.

**Fig 1:**
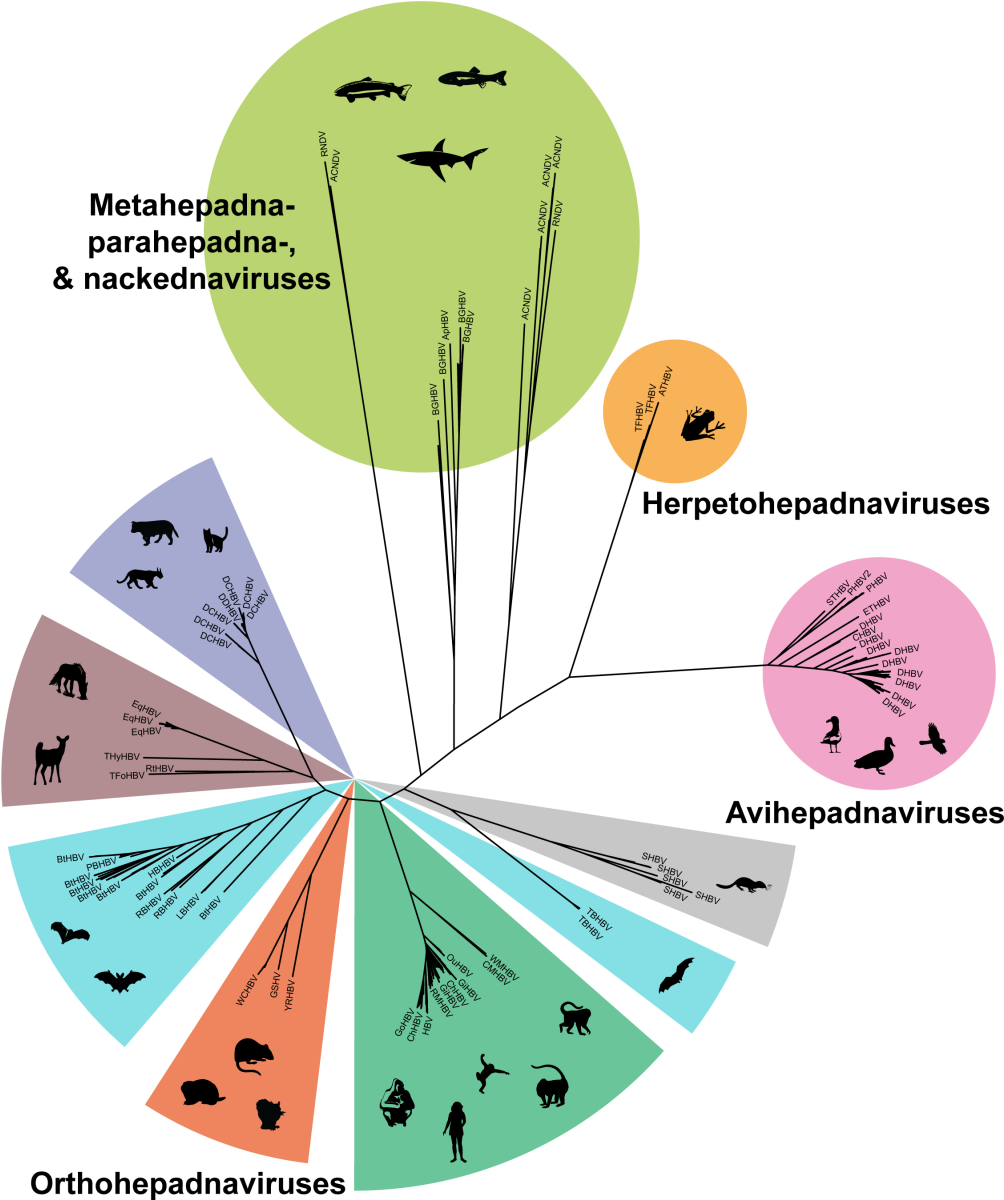
HBV-related hepadnaviruses can be found in a broad range of zoonotic reservoirs. The unrooted phylogenetic tree depicts divergence and diversity among 659 selected hepadnaviruses. The phylogenetic tree includes metahepadnaviruses, parahepadnaviruses, and nackednaviruses (fish), avihepadnaviruses (birds), herpetohepadnaviruses (amphibians and reptiles), and orthohepadnaviruses (mammals). The orthohepadnavirus genus includes dogs, cats, and lynxes; bats; horses, and ringtails; groundhogs, squirrels, and rats; primates; and shrews (from left to right). The genome sequences selected were chosen based on their sequence length similarity to that of human HBV, with the exception of sequences from the metahepadnaviruses, parahepadnaviruses, and nackednaviruses genera that includes shorter sequences.

A defining feature of HBV biology is its remarkably restricted host range. Natural infection is largely limited to humans and chimpanzees (5), underscoring the stringent species-specific requirements for viral entry and replication. Nevertheless, related hepadnaviruses have been identified across a broad spectrum of vertebrate hosts, including fish (6–8), reptiles (9), birds (avihepadnaviruses), and mammals (orthohepadnaviruses) (10–16) (Fig. 1). Among these, duck hepatitis B virus (DHBV) (17) and woodchuck hepatitis virus (WHV) (18) have been particularly instrumental as experimental models, providing key insights into viral replication strategies and pathogenesis (14).

HBV is among the smallest known animal DNA viruses, harboring a compact ~3.2 kb genome with highly constrained coding capacity (19). Its genome is organized into four open reading frames (ORFs), which encode the principal viral gene products. These include (i) the viral polymerase (Pol), a multifunctional enzyme responsible for reverse transcription, and RNA encapsidation(20, 21); (ii) the envelope proteins (HBs), comprising the small (S), medium (M), and large (L) surface antigens that form the viral envelope and mediate receptor engagement and entry; (iii) the core gene products, including the capsid protein (HBc), which assembles into the nucleocapsid and is essential for genome packaging and replication, as well as the secreted hepatitis B e antigen (HBe) derived from the precore region (22–24); and (iv) the regulatory X protein (HBx), a pleiotropic factor that modulates viral transcription and host cell pathways to facilitate infection (25–27).

## THE HBV REPRODUCTIVE CYCLE IN HUMAN HEPATOCYTES

**Fig 2:**
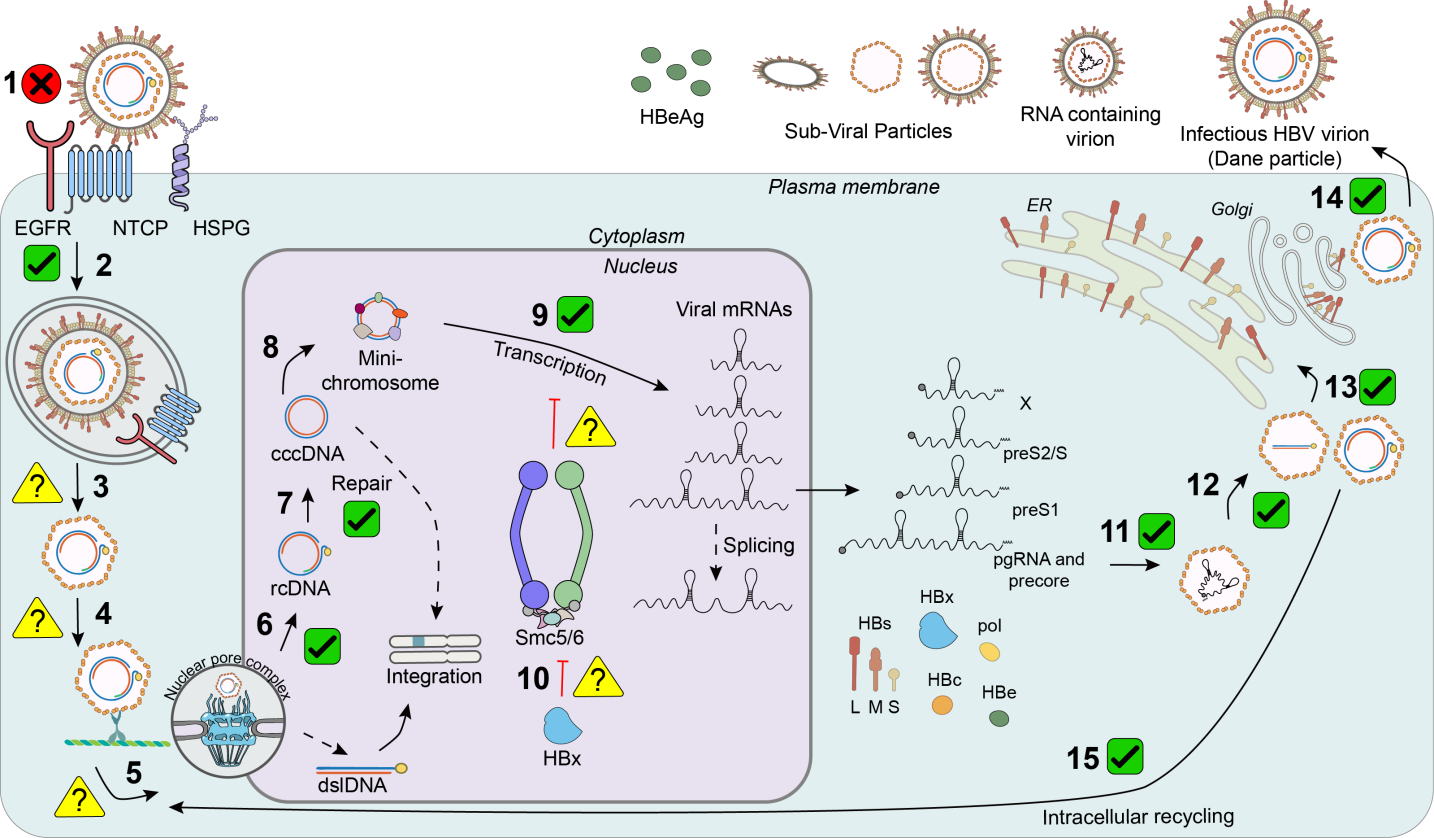
The hepatitis B virus reproductive cycle is blocked at multiple stages in murine cells. Green check marks indicate steps in the viral reproductive cycle that are supported in murine cells. The first block in the viral entry can be overcome by expressing human NTCP in murine cells. The yellow question marks highlight potential barriers. The red crosses indicate known blocks or inefficient processes in murine cells. (1). Virion attachment to HSPG and receptor binding to NTCP. (2) Internalization via receptor-mediated endocytosis. (3) Viral membrane fusion. (4) Microtubule- and dynein-dependent capsid transport to the nucleus. (5) Transport of the nucleocapsid into the nucleus via the nuclear pore complex. (6) Capsid disassembly and rcDNA release. (7) cccDNA formation. (8) Minichromosome formation. (9) Viral gene transcription. (10) HBx-dependent antagonization of Smc5/6-mediated suppression of viral gene transcription. (11) Nucleocapsid assembly. (12) pgRNA is reverse transcribed into rcDNA within the capsid. (13) Envelopment. (14) Virion and subviral particle release. (15) cccDNA replenishment through intracellular recycling pathway.

HBV infection of hepatocytes—the only replicative cellular reservoir—is initiated through a multistep entry process involving sequential attachment and receptor engagement (Fig. 2). Virions first associate with the hepatocyte surface via low-affinity interactions with heparan sulfate proteoglycans (HSPGs), such as glypican-5 (28, 29). This initial attachment facilitates high-affinity binding of the N-terminal domain of the large surface protein (PreS1) to the sodium taurocholate co-transporting polypeptide (NTCP), a bile acid transporter expressed on hepatocytes and the *bona fide* entry receptor for HBV (30, 31). Viral internalization occurs predominantly via clathrin-mediated endocytosis (32) and is further facilitated by host cofactors, including epidermal growth factor receptor (EGFR) and scavenger receptor class F member 2, SCARF2 (33–35).

New evidence suggests that internalized HBV virions are transported within SCARF2-containing endosomal vesicles to the cytoplasmic side of nuclear pore complexes (NPCs), accompanied by ultimate HBV nucleocapsid release from the endosomes for nuclear entry (35). Productive nuclear import requires structural rearrangements or partial disassembly of the capsid to expose nuclear localization signals (NLSs), which engage karyopherin α/β transport pathways and mediate docking at the NPC (36–42).

The incoming nucleocapsid delivers relaxed circular DNA (rcDNA) into the nucleus. This rcDNA genome is structurally unique and contains several lesions: (i) covalent linkage of Pol to the 5′ end of the minus strand via a tyrosyl-phosphodiester bond; (ii) a terminal redundancy region forming a short DNA flap; (iii) a capped RNA primer on the plus strand; and (iv) an incomplete plus strand, resulting in a single-stranded gap. Consequently, rcDNA must be converted into covalently closed circular DNA (cccDNA), the episomal transcriptional template that establishes persistent infection (43).

The formation of cccDNA is a multistep repair process that relies extensively on host DNA repair machinery but does not require the viral polymerase activity (44). This process includes the removal of the covalently linked polymerase (mediated in part by TDP2) (45), excision of RNA and DNA flaps (e.g., via FEN1) (46, 47), completion of plus-strand DNA synthesis by host DNA polymerases (including POLκ, POLδ, POLη, and POLλ) (46, 48–50) in conjunction with PCNA (46) and RFC (46), and ligation of remaining nicks by DNA ligases I and III (46). Additional host factors, such as YBX1 (51), may contribute to this process. Notably, many of these steps likely occur simultaneously *in vivo* and may involve partially redundant host pathways (52).

Once repaired, cccDNA is organized into a chromatinized minichromosome through association with host histones and viral proteins, including HBc and HBx (53, 54). This episome undergoes dynamic epigenetic regulation, including histone modifications that influence transcriptional activity (53, 55). cccDNA serves as the template for all viral transcripts, which are synthesized by host RNA polymerase II from four promoters (preS1, preS2/S, core, and X) and regulated by two enhancers (Enh1 and Enh2).

A key host restriction mechanism targeting cccDNA involves the structural maintenance of chromosomes 5/6 (Smc5/6) complex, which suppresses viral transcription. HBV counteracts this restriction through the action of HBx, which recruits the DDB1–Cullin4 E3 ubiquitin ligase complex to mediate the degradation of Smc5/6 (56, 57). Notably, because HBx is not packaged in virions, its expression depends on initial transcription from newly formed cccDNA, presenting a long-standing “chicken-and-egg” paradox in HBV biology. Recent studies suggest that differential promoter accessibility and chromatinization states, particularly at the X promoter, may facilitate early HBx expression despite Smc5/6-mediated repression (58).

All viral RNAs share a common 3′ polyadenylation site and are 5′ capped, reflecting their synthesis by host RNA polymerase II. Among these transcripts, the pregenomic RNA (pgRNA) serves both as the template for reverse transcription and as the mRNA for core and polymerase proteins. Encapsidation of pgRNA is initiated by binding of Pol to the ε stem-loop structure on pgRNA, triggering nucleocapsid assembly. Within the capsid, pgRNA is reverse transcribed through a series of coordinated steps, including primer translocation and DNA strand synthesis, ultimately yielding rcDNA genomes (59).

Mature nucleocapsids follow one of two fates: they can acquire an envelope containing viral surface proteins and be secreted as infectious virions, or they can be redirected to the nucleus to replenish the cccDNA pool via intracellular amplification. This recycling pathway complements *de novo* cccDNA formation from incoming virions and contributes to the persistence of infection (60, 61).

Virion secretion occurs via the host secretory pathway and involves budding into multivesicular bodies. In addition to infectious Dane particles, HBV-infected cells release large quantities of non-infectious subviral particles composed of HBsAg (spheres and filaments), as well as soluble HBeAg and naked capsids. These excess viral products are thought to contribute to immune evasion by acting as decoys for host immune responses (2, 60).

Although HBV DNA can integrate into the host genome, this process is not required for viral replication. While these integrants may remain transcriptionally active, they appear dispensable for the completion of the HBV replicative cycle (62). Instead, the persistence of chronic infection is primarily sustained by the remarkable stability and self-amplifying capacity of the cccDNA minichromosome, which serves as the central reservoir for viral gene expression and represents a major barrier to achieving a cure.

## HBV HAS A NARROW SPECIES TROPISM

Much of our current understanding of HBV molecular biology has been informed by studies of related hepadnaviruses, particularly duck hepatitis B virus (DHBV) (59). While these surrogate systems have been invaluable for dissecting fundamental aspects of the viral life cycle, they incompletely capture the complex interplay between HBV and its natural mammalian host. This limitation has been a major barrier to understanding HBV pathogenesis *in vivo* and to the development of curative therapies.

An ideal animal model for HBV would be fully susceptible and permissive to infection with authentic HBV, recapitulate key features of human disease, support all viral genotypes, and possess an intact and manipulable immune system. In addition, such a model would be experimentally tractable, cost-effective, and amenable to longitudinal analysis. To date, no single system fulfills all of these criteria.

Historically, chimpanzees represented the gold standard for HBV research due to their natural susceptibility to infection and ability to recapitulate many aspects of human disease (63). However, their use is now severely restricted due to ethical considerations, limited availability, and high costs (7). Other naturally occurring hepadnaviruses have been identified in species such as woodchucks (WHV) (18), Beechey ground squirrels (12, 15, 16), and woolly monkeys (11) (Fig. 1), and these models have provided important insights into viral replication and hepatocarcinogenesis. Nevertheless, genetic and antigenic differences between these viruses and HBV limit their utility for studying species-specific aspects of HBV biology and for evaluating therapeutic strategies.

Rodents would represent the most practical experimental system due to their low cost, rapid breeding, and extensive genetic toolkit. However, mice and rats are not naturally susceptible to HBV infection. While transgenic (64, 65) and hydrodynamic injection-based (66) approaches have enabled expression of HBV genomes in murine hepatocytes—facilitating studies of viral gene expression, immune responses, and pathogenesis—these models bypass critical early steps of the viral life cycle and, therefore, do not faithfully recapitulate natural infection.

## HOST RANGE RESTRICTION OF HBV IN RODENTS

The identification of the NTCP as the entry receptor for HBV and hepatitis D virus (HDV) raised the possibility that species restriction could be overcome at the level of viral entry. Indeed, ectopic expression of hNTCP in murine hepatocytes permits HBV binding and internalization *in vitro* (31, 67) and *in vivo* (68–70). However, productive infection does not ensue, indicating that additional post-entry barriers critically limit the HBV life cycle in mouse cells (Fig. 2).

As detailed above following entry into human hepatocytes, nucleocapsids are transported to the nucleus, where the rcDNA genome is converted into transcriptionally active cccDNA. In murine systems, there is limited evidence that this process occurs efficiently. In HBV transgenic mice, viral RNAs are transcribed from integrated HBV DNA, but cccDNA is rarely detectable (71). Nonetheless, these animals produce infectious virions capable of infecting chimpanzees (72), demonstrating that downstream processes such as capsid assembly and virion secretion are largely intact in mouse cells.

This phenotype suggests that species-specific restriction operates upstream of or at, the level of cccDNA formation and/or maintenance. Several lines of evidence support this notion. First, heterokaryon experiments combining murine hepatoma cells expressing hNTCP with human hepatoma cells restore susceptibility to HBV infection, indicating that murine cells lack one or more essential human-specific factors required for productive infection (73). Second, while cccDNA formation has been reported under certain experimental conditions in murine hepatoma cell lines (e.g., certain AML12 subclones) (74, 75) or in HBV transgenic mice lacking hepatocyte nuclear factor 1 (HNF1) (71), these events occur at low efficiency, suggesting that the restriction is not absolute but rather reflects suboptimal execution of one or more steps in the pathway.

A central unresolved question is which step(s) between viral entry and cccDNA establishment are impaired in murine cells. The processes required for cccDNA formation include nucleocapsid trafficking, capsid disassembly, nuclear import of rcDNA, and rcDNA repair. Biochemical reconstitution assays and studies using rcDNA mimics have demonstrated that murine nuclear extracts—and even purified murine orthologs of key DNA repair enzymes—are capable of supporting rcDNA repair and cccDNA formation (76, 77). These findings are further supported by studies in murine cells expressing HBV pgRNA, where intracellular rcDNA-containing capsids can be generated and cccDNA becomes detectable under these specific experimental conditions. However, results obtained under authentic infection conditions suggest a more complex situation. Although rcDNA can be detected in the nuclei of murine hepatocytes expressing human NTCP following HBV infection, cccDNA remains undetectable or is present only at extremely low levels (75). Similarly, HBV transgenic mouse models generally do not exhibit robust authentic cccDNA formation despite substantial HBV transgene expression and viral replication activity (64, 78). While cccDNA has been reported in AAV-HBV mouse models (79), subsequent studies suggested that at least part of this signal may arise from intramolecular recombination of overlength HBV transgene constructs rather than *bona fide* rcDNA-to-cccDNA conversion (80).

Accumulating evidence points to earlier steps in the pathway. Comparative studies of HBV infection in human and murine hepatocytes reveal that, although rcDNA can be generated following intracellular amplification or experimental delivery, cccDNA formation is largely restricted to human cells (77). Notably, rcDNA delivered as naked DNA or synthetically generated cccDNA templates can be transcriptionally active in murine hepatocytes (76), demonstrating that once cccDNA is formed, downstream transcriptional processes are functional. Although transcriptional differences cannot be completely ruled out, current evidence suggests HBV gene transcription is not the primary determinant of host range restriction as Smc5/6 antagonism by HBx appears to be an evolutionarily conserved function of HBV in mammals (81) but definitive proof using infectious culture system and murine models is missing. This further supports the conclusion that the principal block lies upstream of cccDNA formation.

Additional studies suggest that inefficient nucleocapsid disassembly represents a critical species-specific barrier to HBV infection in murine cells (76, 77). In mouse cells, cytoplasmic nucleocapsids exhibit increased stability and resistance to nuclease digestion compared with those in human hepatocytes, consistent with a failure to undergo the partial disassembly required for rcDNA release and subsequent nuclear import (77). In contrast, in human hepatocytes, a subset of nucleocapsids containing mature rcDNA adopts a destabilized conformation that facilitates genome release. Together, these observations implicate species-specific differences in capsid dynamics and uncoating as important determinants of HBV host restriction (77). A recent study, however, challenged this model and instead suggested the existence of a restriction at a late/post-entry stage of infection (82) . Expression of HBV pgRNA from a CMV promoter in murine cells supported viral replication and cccDNA formation through intracellular amplification, a pathway that requires nucleocapsid uncoating and nuclear delivery of rcDNA, analogous to events occurring during *de novo* infection. These findings, together with the recent discovery that human SCARF2 functions as an intracellular HBV receptor (35), raise the possibility that species-specific differences in the EGF-like domain 4-6 of SCARF2 reported to interact with preS1 contribute to the observed restriction by impairing intracellular trafficking of incoming virions and/or their escape from endosomal compartments. Nevertheless, the apparent discrepancy between these findings and earlier studies may reflect differences in the experimental systems used to interrogate HBV entry and post-entry events.

Collectively, these findings support a model in which HBV entry into murine hepatocytes can be (partially) achieved through ectopic expression of hNTCP, but subsequent late/post-entry steps remain inefficient. In particular, nucleocapsid uncoating and/or efficient delivery of rcDNA to the nuclear DNA repair machinery appear to be suboptimal. Thus, although the intrinsic capacity to repair rcDNA into cccDNA may be largely conserved in murine cells, insufficient substrate availability resulting from defective upstream processing likely limits efficient cccDNA establishment during authentic infection.

These apparent blocks may not, *per se,* be conserved across all rodent species as recent work suggests that hamster cells can support the entire HBV reproductive cycle when human or a humanized version of hamster NTCP is expressed (83). Nonetheless, at present, evidence is missing that overcoming the block at the level of entry would suffice to establish HBV infection in hamsters.

Another layer of restriction may reside in species-specific differences in cell-intrinsic antiviral immunity. HBV has long been described as a “stealth virus,” a concept largely derived from studies in human and chimpanzee hepatocytes where innate immune activation following infection is minimal, delayed, or otherwise attenuated (84, 85). However, this characterization may be highly host specific. In non-human species, HBV may be more readily detected by innate immune sensors, resulting in stronger antiviral responses that restrict completion of the viral life cycle. Although HBV has evolved multiple mechanisms to dampen innate immune signaling in human cells—including activities mediated by HBx and the viral polymerase targeting pathways involving MAVS (86), STING (87), and DDX3 (88)—these immune evasion strategies are likely adapted to human host factors and may function less efficiently in other species. Consequently, species-specific differences in innate immune sensing, antiviral signaling, and downstream restriction pathways may constitute an additional post-entry barrier contributing to the exceptionally narrow host range of HBV.

Elucidating the molecular basis of these species-specific differences remains a critical goal, as overcoming these barriers would enable the development of fully immunocompetent small animal models for HBV infection.

## HBV RESTRICTION IN PRIMATES

Beyond rodent models, host restriction mechanisms also shape susceptibility in primates, as HBV displays a strikingly narrow host range even within this group, with robust infection largely limited to humans and chimpanzees. The chimpanzee model has been instrumental in defining the natural course of HBV infection, including viral replication dynamics, host immune responses, and the pathological consequences of persistent infection such as liver inflammation and hepatocellular carcinoma (63). However, the use of chimpanzees is now severely restricted (89), and more commonly used non-human primates (NHPs), including rhesus macaques (RMs) and cynomolgus macaques, are not permissive to HBV infection.

This restriction can be largely attributed to species-specific differences in the amino acid (AA) sequence of the HBV entry receptor, NTCP (90–92). Structural and functional analyses have identified residues within positions 84–87 and 157–165 of human NTCP (hNTCP) as critical determinants for viral entry, with residue 158 emerging as a key contributor. These regions map to functionally distinct domains of NTCP: residues 84–87 are located within an extracellular loop mediating interaction with the preS1 domain of the HBV envelope, whereas residues 157–165 form part of the bile acid transport tunnel that also serves as a docking site for preS1 (93).

Comparative and mutational analyses of NTCP orthologs from Old World and New World monkeys have provided further mechanistic insight into this restriction. Introduction of humanizing substitutions, particularly at residue 158 (R158G), enables binding of preS1-derived peptides to macaque NTCP, whereas the reciprocal mutation disrupts binding in permissive species (94). Additional substitutions, including combinations at positions 158 and 165, further enhance viral binding and entry (91). Similarly, in New World monkey NTCP, residues within the 84–87 region contribute to susceptibility, with mutations at positions 84 and 87, and to a lesser extent 86, conferring the ability to support HBV uptake and downstream infection, as evidenced by cccDNA formation and sensitivity to entry inhibition (91). These findings underscore that relatively subtle sequence variation in NTCP is sufficient to impose a stringent species barrier at the level of viral entry.

Consistent with this notion, ectopic expression of hNTCP in rhesus macaques via adenovirus-, adeno-associated virus-mediated delivery (95, 96) or transgenic expression (97) permits HBV entry and results in acute, transient infection in immunocompetent animals, which can progress to persistent infection under conditions of immunosuppression (95). These observations establish that overcoming the entry barrier is sufficient to enable at least partial viral replication in small NHPs although additional host factors likely limit the efficiency and durability of infection.

Complementary approaches have explored whether viral adaptation can overcome these host restrictions (91). In this context, WMHBV, a related hepadnavirus infecting New World primates, provides an informative intermediate. WMHBV is capable of infecting primary marmoset hepatocytes, and replacement of the N-terminal 48 amino acids of the HBV preS1 domain with the corresponding WMHBV sequence generates a chimeric virus that infects these cells *in vitro* with moderate efficiency. These findings demonstrate that relatively limited changes in the viral envelope can expand host range, providing proof-of-concept that HBV’s species restriction is, at least in part, surmountable through viral adaptation.

Together, these studies highlight NTCP as a primary determinant of HBV tropism in primates, while also revealing that both host receptor variation and viral envelope plasticity contribute to the stringent, yet potentially malleable, species barrier.

## HBV SURROGATE MODELS

Although the restricted host range of HBV limits infection even in closely related primates, hepadnaviruses themselves are widely distributed across vertebrate species (Fig. 1). Only a limited subset of these viruses, however, has been adapted into experimental systems to model HBV biology *in vivo*. In addition to rodents and non-human primates, surrogate models based on related hepadnaviruses–most notably those infecting woodchucks (WHBV), domestic ducks (DHBV), and New World primates such as the woolly monkey (WMHBV)–have been characterized to study viral infection in species where HBV itself does not readily establish infection. These systems, while not fully recapitulating HBV infection, provide complementary comparative frameworks to interrogate the host and viral determinants that shape hepadnavirus tropism.

Among these, WHBV infection in *Marmota* species remains the most extensively characterized. WHBV shares key virological features with HBV, including genome organization and replication strategy, with approximately 60%–70% nucleotide sequence similarity (18). Infection in woodchucks recapitulates major aspects of HBV-associated liver disease, including progression from acute infection to chronic hepatitis and hepatocellular carcinoma. As in humans, infection outcome is strongly age-dependent, with neonatal infection frequently resulting in persistence, whereas adult infection is typically self-limited. Despite these similarities, important differences exist, including distinct integration patterns (98) and host-specific features that must be considered when extrapolating findings to human HBV infection.

Duck hepatitis B virus (DHBV) represents a more evolutionarily distant model that has nonetheless been central to defining fundamental aspects of the hepadnavirus life cycle (17). Studies in duck hepatocytes and *in vivo* infection systems have enabled detailed characterization of viral replication, including reverse transcription and cccDNA formation (59). However, DHBV differs substantially from HBV, sharing only ~40% sequence homology and utilizing a distinct cellular receptor for entry, carboxypeptidase D (DCPD) (99), underscoring key differences in host–virus interactions.

The woolly monkey hepatitis B virus (WMHBV) provides a non-human primate-associated hepadnavirus that occupies an intermediate phylogenetic position (11). Although direct studies in woolly monkeys are limited, WMHBV infection has been examined in related species, including spider monkeys (100) and squirrel monkeys (101). These models highlight species-specific differences in susceptibility, with infection typically resulting in transient viremia and immune-mediated clearance, although persistence can be achieved under certain experimental conditions.

Tupaias (tree shrews) represent a unique non-primate model that is phylogenetically closer to primates than rodents and is experimentally susceptible to HBV infection. Infection in tupaias reproduces some features of human disease, including age-dependent progression and similar liver pathology (102). Primary tupaia hepatocytes are also permissive to HBV and related viruses *in vitro* (102, 103). However, broader application of this model is limited by genetic heterogeneity, relatively low viral titers *in vivo*, and the scarcity of species-specific reagents.

More recently, additional hepadnaviruses have been identified in species such as domestic cats (13), dogs (104), horses (105), and bats (10), further expanding the known diversity of the family. For example, domestic cat hepadnavirus (DCHBV) shares key genomic and organizational features with HBV, and while primary feline hepatocytes have been shown to support DCHBV infection (106), experimental infection models in cats have not yet been established (107). Notably, the expression of hNTCP in pig hepatocytes appears sufficient to overcome the major barrier to productive HBV infection, highlighting the potential of transgenic pigs as a relevant model system (92, 103). As these newly identified viruses are further characterized, they may provide additional comparative systems to better delineate the molecular and cellular barriers that enforce the remarkably narrow host range of HBV.

## CONCLUSIONS AND OUTLOOK

Despite decades of intensive research, the development of an immunocompetent, fully permissive animal model of chronic HBV infection remains an unresolved challenge. While significant progress has been made, currently available systems—including genetically engineered models and surrogate hepadnavirus infections—only partially recapitulate key aspects of HBV biology and pathogenesis.

Genetic and vector-based approaches have provided important experimental entry points. Transgenic expression of the HBV genome or delivery via viral vectors such as adeno-associated virus (AAV) or adenovirus can overcome early restrictions and enable robust viral gene expression in hepatocytes (64, 108). However, these systems do not establish a *bona fide* infection, as the viral life cycle is incomplete and infectious progeny are unable to spread to naïve cells. As such, they model intracellular replication and antigen expression rather than viral transmission dynamics.

Humanized xenotransplantation models offer a complementary strategy by enabling HBV infection *in vivo* within a human hepatocyte context (109–111). Advances in liver chimeric mouse systems, including improved engraftment efficiency and the use of *in vivo*-expanded or stem cell-derived hepatocytes, have increased experimental flexibility and scalability while reducing donor-dependent variability (reviewed in reference 112). Nevertheless, current models remain limited by the absence of a fully humanized hepatic microenvironment, as non-parenchymal cell populations critical for intercellular crosstalk are largely of murine origin. Moreover, the profound immunodeficiency required for stable engraftment restricts the ability to interrogate antiviral immune responses. Efforts to co-engraft components of a human immune system have begun to address this limitation, and such dual humanization approaches have demonstrated partial immune activation and virus-associated pathology, but these systems remain technically demanding and incompletely representative (113–115).

Collectively, these limitations underscore a central barrier in the field: the lack of accessible, immunocompetent models that faithfully support chronic HBV infection. Existing systems are constrained by species-specific entry and post-entry blocks, by incomplete viral life cycles, or by reliance on surrogate viruses in non-standard host species. These shortcomings complicate the interpretation of preclinical findings and limit their translational relevance. The development of tractable, immunocompetent models—ideally in both small animals and non-human primates—would enable more physiologically relevant studies of viral persistence, immune control, and therapeutic intervention. Such systems would also facilitate a tiered experimental pipeline, in which hypotheses can be tested at scale in small animals and subsequently validated in higher-order models.

Beyond model development, reducing the global burden of HBV will continue to rely on effective public health measures, including vaccination, screening, and efforts to reduce disease-associated stigma. However, these strategies do not address the large population of individuals living with chronic infection. The existence of rare cases of functional cure provides compelling evidence that durable viral control is achievable and underscores the need for continued innovation in therapeutic development.

Achieving this goal will require coordinated, sustained efforts across the field. Model development must be guided by clearly defined use cases and adhere to rigorous standards of reproducibility. Broad access to reagents and model systems will be essential to accelerate progress and enable cross-validation. Continued investment from both public and private sectors is critical to support the long timelines and technical complexity inherent to this work. Equally important is the preservation of a research environment that enables scientifically grounded and ethically sound approaches, including access to critical biological materials where appropriate. Finally, close integration between academia and industry, coupled with efficient data sharing, will be essential to translate advances in basic virology into effective therapies.

Together, these efforts will be required to overcome the persistent barriers imposed by HBV host restriction and to ultimately enable the development of curative strategies for chronic hepatitis B.
